# Reading comprehension differences between children with Autism Spectrum Disorder and low cognitive abilities and children with Autism Spectrum Disorder and intact cognitive skills: the roles of decoding, fluency and morphosyntax

**DOI:** 10.3389/fpsyg.2024.1357590

**Published:** 2024-04-10

**Authors:** Eleni Peristeri, Christos A. Frantzidis, Maria Andreou

**Affiliations:** ^1^Department of English Studies, Aristotle University of Thessaloniki, Thessaloniki, Greece; ^2^School of Computer Science, University of Lincoln, Lincoln, United Kingdom; ^3^Department of Speech and Language Therapy, University of Peloponnese, Kalamata, Greece

**Keywords:** Autism Spectrum Disorder, intellectual functioning, reading comprehension, decoding, fluency, morphosyntax, metalinguistic awareness

## Abstract

**Introduction:**

Reading comprehension is one of the most important skills learned in school and it has an important contribution to the academic success of children with Autism Spectrum Disorder (ASD). Though previous studies have investigated reading comprehension difficulties in ASD and highlighted factors that contribute to these difficulties, this evidence has mainly stemmed from children with ASD and intact cognitive skills. Also, much emphasis has been placed on the relation between reading comprehension and word recognition skills, while the role of other skills, including fluency and morphosyntax, remains underexplored. This study addresses these gaps by investigating reading comprehension in two groups of school-aged children with ASD, one with intact and one with low cognitive abilities, also exploring the roles of word decoding, fluency and morphosyntax in each group’s reading comprehension performance.

**Methods:**

The study recruited 16 children with ASD and low cognitive abilities, and 22 age-matched children with ASD and intact cognitive skills. The children were assessed on four reading subdomains, namely, decoding, fluency, morphosyntax, and reading comprehension.

**Results:**

The children with ASD and low cognitive abilities scored significantly lower than their peers with intact cognitive abilities in all reading subdomains, except for decoding, verb production and compound word formation. Regression analyses showed that reading comprehension in the group with ASD and intact cognitive abilities was independently driven by their decoding and fluency skills, and to a lesser extent, by morphosyntax. On the other hand, the children with ASD and low cognitive abilities mainly drew on their decoding, and to a lesser extent, their morphosyntactic skills to perform in reading comprehension.

**Discussion:**

The results suggest that reading comprehension was more strongly affected in the children with ASD and low cognitive abilities as compared to those with intact cognitive skills. About half of the children with ASD and intact cognitive skills also exhibited mild-to-moderate reading comprehension difficulties, further implying that ASD may influence reading comprehension regardless of cognitive functioning. Finally, strengths in decoding seemed to predominantly drive cognitively-impaired children’s reading performance, while the group with ASD and intact cognitive skills mainly recruited fluency and metalinguistic lexical skills to cope with reading comprehension demands, further suggesting that metalinguistic awareness may be a viable way to enhance reading comprehension in ASD.

## 1 Introduction

Autism Spectrum Disorder (ASD) is a neurodevelopmental condition marked by challenges in various domains ranging from children’s social and communication skills to their cognitive and language abilities. Especially language ability varies widely for children with ASD (Tager-Flusberg et al., 2005; Peristeri et al., 2017; Andreou et al., 2020) with pragmatic language difficulties (i.e., language use in context) being a hallmark feature even for high-functioning children whose IQ lies in the average range (Baron-Cohen, 1988; Peppé et al., 2006; Lam and Yeung, 2012; Andreou and Skrimpa, 2020; Peristeri and Tsimpli, 2022; Lampri et al., 2023). Among verbally-able children with ASD, language difficulties have been reported to have a cascading negative effect on children’s academic success leading to underachievement as compared to typically-developing (TD) peers. There is significant variability in the academic achievements of school-aged children with ASD (Keen et al., 2016); some perform as expected, given their symptomatology and IQ, and might perform better in some tasks such as visual reasoning (Mayes and Calhoun, 2003). Many children with ASD, however, perform worse academically than their IQ would predict (Manti et al., 2011; Kim et al., 2018; Peristeri and Andreou, 2024). For the children with ASD and high IQ, the variability in academic achievement is even higher, since some perform well in mainstream schools, while others need to attend special education settings (Church et al., 2000). Among academic skills, reading comprehension, i.e., making sense of what is read, is a noted area of challenge for children with ASD. Though reading comprehension skills have been investigated in populations with ASD, studies have mainly focused on the children’s word decoding and oral language abilities as being the most important predictors contributing to high vulnerability for poor reading comprehension outcomes (Cronin, 2014; Solari et al., 2017; Davidson, 2021). Reciprocal relationships between reading comprehension outcomes and individual variation in other academic skills have received less attention. Moreover, there is little research on the effects of non-verbal cognitive ability on reading comprehension in ASD, though findings seem to converge toward low reading skills co-occurring with low intelligence (Åsberg et al., 2019; Wang et al., 2022). Previous research on reading comprehension skills in ASD appears to focus heavily on high-functioning individuals with ASD (Solari et al., 2019; Engel and Ehri, 2021; also see the recent meta-analysis by Sorenson Duncan et al., 2021) with little consideration of children with low cognitive abilities, thus, resulting in limited understanding of the generalizability of their findings to children with ASD and low cognitive functioning skills. The current study addresses these gaps by investigating the role of word decoding, fluency, morphosyntax and intellectual functioning in the reading comprehension skills of children with ASD and intact or low cognitive abilities using standardized assessment tests for all the aforementioned domains.

Reading comprehension in typical development appears to be a multicomponent operation that is shaped by individual differences in language ability. The decoding component (also known as visual word recognition component) has been shown to account for a considerable amount of variability in TD children’s reading comprehension development, with earlier achievement at word recognition accuracy leading to larger gains in text comprehension (Nunes et al., 2012; Karageorgos et al., 2020). Also, the contribution of listening comprehension to reading has been mainly conceptualized in terms of the children’s vocabulary and morphosyntax (Adlof and Catts, 2015), both being significant predictors of TD children’s reading comprehension (Ouellette, 2006; Cain and Oakhill, 2014; Siu et al., 2016; Kim et al., 2020). This suggests that children with higher semantic word knowledge and ability to parse syntactically complex structures, which are often encountered in written (rather than oral) language, are better comprehenders as compared to TD peers with lower vocabulary or/and morphosyntactic skills. Finally, several studies provide evidence that morphological metalinguistic awareness is an important factor in reading comprehension in TD children as the ability to understand morphologically complex words boosts word recognition accuracy and speed (Carlisle, 2000; Deacon and Kirby, 2004; Nagy et al., 2006; Ramirez et al., 2014).

Most psycholinguistic studies in reading in ASD highlight a dissociation between word decoding and reading comprehension skills, which is reflected in a discrepancy between advanced decoding and weaker comprehension ability (Minshew et al., 1994; O'Connor and Hermelin, 1994; Mirenda and Erickson, 2000; O'Connor and Klein, 2004; Nation et al., 2006; Huemer and Mann, 2010; Norbury and Nation, 2011; Williamson et al., 2012; Vale et al., 2022). Nation et al. (2006) showed that adolescents with ASD had better word reading skills relative to listening comprehension. Solari et al. (2017) also found that word reading predicted reading comprehension more than listening comprehension in a group of high-functioning individuals. Research has yet to replicate these findings with low-functioning individuals.

Reading words is accomplished through a combination of applying learned letter to sound consistencies (i.e., orthography to phonology mappings) and applying knowledge of word meaning (i.e., semantics). For example, orthography-phonology mappings are sufficient to read consistent words like, “hash” and “cash”; however, semantics is useful for reading inconsistent words like “wash” or “yacht.” In contrast, for pseudowords, i.e., pronounceable but meaningless nonwords, like “fash,” readers only have the resource of orthography-phonology mappings to pronounce the novel letter strings. Thus, readers are at a disadvantage reading pseudowords compared to words (e.g., Share, 2004; de Jong and Messbauer, 2011). Prior studies suggest that children with ASD can be particularly proficient in applying orthography-phonology mappings, which may contribute to the incidence of hyperlexia in this population (Atkin and Lorch, 2006; Zuccarello et al., 2015). This strength may also allow this population to overcome the disadvantage of novelty when reading pseudowords. Crucially, the proficiency of individuals with ASD in applying orthography to phonology mappings has been shown to be underpinned by the recruitment of the right-hemisphere homolog of the visual word form area besides the expected left-lateralized activation in the left-hemisphere ventral visual stream that is commonly observed in word reading in TD children (Graves et al., 2022; McCabe, 2023).

In contrast to word and pseudoword decoding skills that appear to be relatively spared or even superior in children with ASD, higher-order reading skills seem to be disproportionally more impaired in the specific population. School-aged children with ASD are at risk of persistent reading comprehension difficulties, with challenges reported in over 50% of students (Mayes and Calhoun, 2007; Whitby and Mancil, 2009; Åsberg et al., 2019, among others). McIntyre et al. (2017) found that in a sample of children with ASD, Attention Deficit Hyperactivity Disorder, and TD children, 55, 33, and 11%, performed one standard deviation or greater below the mean on a standardized measure of reading comprehension, respectively. Reading comprehension in ASD has shown strong correlations with both word decoding accuracy and listening comprehension skills [Kim et al., 2018; Åsberg et al., 2019; Knight et al., 2019; Solari et al., 2019; also see Sorenson Duncan et al.’s (2021) recent meta-analysis for similar conclusions], consistent with the Simple View of Reading framework that supports that reading comprehension draws broadly on both visual word recognition and oral competence skills in children (Hoover and Gough, 1990). Besides word decoding and listening comprehension as critical components of reading comprehension in ASD, the role of morphosyntax has received little attention. Åsberg et al. (2019) found that poor readers on the spectrum had low syntactic language skills, while other studies (Jacobs and Richdale, 2013; Ricketts et al., 2013) have found that syntax was a significant predictor of reading comprehension in ASD, though of weaker magnitude relative to vocabulary (Davidson et al., 2018). More recent studies (McIntyre et al., 2020; Peristeri and Tsimpli, 2020) hypothesize that reading comprehension difficulties in ASD partially stem from their pragmatic difficulties that leads to a degradation in their inferencing skills, including the coherent organization of categories and events, which in turn influences the degree to which pragmatically relevant information can be retrieved and used in order to make sense of words and sentences in a text in real time.

Though research suggests that word decoding skills have an important contribution to these children’s reading comprehension performance, the relations between reading comprehension and additional ability domains that have been shown to be important in the prediction of reading comprehension in TD children, such as fluency, i.e., reading a text with both speed and accuracy (Bourassa et al., 1998; Fuchs et al., 2001), vocabulary knowledge (Nation and Snowling, 1998; Muter et al., 2004; Protopapas et al., 2007) and morphosyntactic skills (Adlof and Catts, 2015; Gottardo et al., 2018), still remain underexplored in ASD. Moreover, though previous research has documented that intellectual functioning skills influence reading skills in TD children (Corso et al., 2016; Johann et al., 2020), our knowledge of reading comprehension skills in children with ASD and low cognitive abilities is still limited.

Extracting information from written material is a pillar of academic success (Oakhill and Cain, 2007; García-Madruga et al., 2014). For most children with ASD, making sense of what they read is a noted area of challenge. To support these children’s academic progress, we need to gain a deeper understanding of the component skills contributing to their reading comprehension difficulties. The current study seeks to enhance our understanding of the domains that influence these children’s ability to understand what they read, further considering the influence of general cognitive functioning on the children’s reading comprehension abilities. Specifically, the study explores the relations between reading comprehension, word and pseudoword decoding, and morphosyntax in two groups of children with ASD, one with intact and one with low cognitive skills, to investigate plausible pathways for the contribution of each of these domains to reading comprehension outcomes in each group.

## 2 Method

### 2.1 Participants

The study included in total 38 (27 males, 11 females) Greek-speaking children with ASD that were split in two groups, namely, Group A (mean age: 10; 7) comprising 22 (18 males, 8 females) children with intact cognitive skills (Full-Scale IQ (FSIQ) > 80), and Group B (mean age: 10; 3) comprising 16 children (9 males, 3 females) with low cognitive skills (FSIQ <70) (see Table 1). The two groups did not differ in age, *F*(1, 36) = 1.149, *p* = 0.291, *η^2^* = 0.03. The children were recruited from the geographical region of Macedonia in northern Greece, and were referred by Centers for Differential Diagnosis, Assessment, Counseling and Evaluation (KEDASY) that constitute the official state centers responsible for the diagnosis and assessment of ASD and other developmental disorders in Greece. All children received a formal clinical diagnosis of ASD at preschool age at KEDASY on the basis of the DSM-V, and ICD-10 criteria (World Health Organization, 1993; American Psychiatric Association, 2013), a record review conducted by teams with diverse expertise (psychiatrist, clinical psychologist, specialized educator, social worker, speech language pathologist), as well as the Autism Diagnostic Interview-Revised (ADI-R; Lord et al., 1994). The groups included 3rd, 4th, 5th and 6th graders in public primary schools, and 1st graders in lower-secondary schools in Greece. All children attended mainstream classes. Children’s verbal IQ (VIQ), Performance (PIQ) and FSIQ scores were estimated using the Greek version of the Wechsler Intelligence Scale for Children, 5th Edition (WISC-V GR; Wechsler, 2014; Greek version by Stogiannidou et al., 2017). Table 1 below presents the mean age and the IQ scores of each group. Group A had significantly higher IQ scores than Group B across all three indices (VIQ: *F* (1, 36) = 79.954, *p* < 0.001, *η^2^* = 0.69; PIQ: *F* (1, 36) = 36.144, *p* < 0.001, *η^2^* = 0.51; FSIQ: *F* (1, 36) = 74.124, *p* < 0.001, *η^2^* = 0.68). All study procedures were approved by the Aristotle University of Thessaloniki Institutional review board (IRB).

**Table 1:** Descriptive statistics (Means, *Standard Deviations*, Ranges) of the ages, VIQ, PIQ and FSIQ performances of the two groups of children with ASD.

	Group A (*n* = 22)	Group B (*n* = 16)
Age (y;m)	10;7 (*1.4*) 8;2–12;7	10;3 (*1.3*) 8;2–12;5
VIQ	93.1 (*11.1*) 80–120	61.6 (*8.3*) 50–70
PIQ	94.4 (*12.9*) 80–127	68.2 (*4.5*) 63–70
FSIQ	96.1 (*12.6*) 80–120	62.9 (*8.3*) 48–70

ASD = Autism Spectrum Disorder; Group A = children with ASD and intact cognitive abilities; Group B = children with ASD and low cognitive abilities; y;m = years;months; VIQ = Verbal IQ; PIQ = Performance IQ; FSIQ = Full-Scale IQ. The numbers in the parentheses indicate the Standard Deviations.

### 2.2 Materials

Data in the current study have been collected from Panteliadou and Antoniou’s (2007) Reading test Alpha (Test-A), which is a standardized psychometric diagnostic tool that has been developed to assess reading abilities in Greek-speaking children aged 8–15 years and identify possible reading difficulties. The test evaluates four reading subdomains, namely, (1) decoding, (2) fluency, (3) morphosyntax, and (4) reading comprehension. In this tool the four subdomains are treated as independent reading skills. Test– retest reliability for all tasks in the test ranges between 0.74 and 0.87. Test-A scores can be converted into percentile scores. A percentile score serves as an index of the percentage of TD children expected to obtain a score equal to or below that obtained by a child. For example, if a child scores at the 5th percentile, it means that only 5% of TD children are expected to obtain a score equal to or below that score; and, conversely, that 95% of TD children are expected to obtain a higher score. According to the test’s instructions, having a score below the 10th percentile is adopted as a cut-off for severe reading difficulties, while scores between the 11th and 30th percentile are adopted as cut off points for moderate reading difficulties. More information on the stimuli and the procedure of the tests assessing each of the four reading domains is provided below.

#### 2.2.1 Decoding

Word decoding skills are assessed through three tests: pseudoword reading; real word reading; and lexical decision on real words and pseudowords (36 items).

In pseudoword reading, the child is asked to read aloud a printed list of 24 pseudowords (mean number of letters: 9.6, *SD*: 3.1) of graded difficulty defined in terms of length, position and number of consonant clusters, and stress mark. In real word reading, the child is asked to read aloud a printed list of 53 real words (mean number of letters: 10.5, *SD*: 3.3). In both pseudoword and real word reading tests, the child receives 1 point for each item being correctly read, so the total accuracy score is 24 and 53, respectively. Testing discontinues when the child makes 5 consecutive errors. In the lexical decision test, the child is asked to read silently rows of intermixed 16 pseudowords and 20 real words, and is asked to identify the real words only. The child receives 1 point (a) for the correct identification of a real word, and (b) for each pseudoword not being identified as a real word, so the total accuracy score is 36. As the test proceeds, the number of items per row increases (rows of three, four and five items). Testing in lexical decision discontinues when the child fails to identify a single real word in three consecutive rows. Scores in pseudoword reading, real word reading and lexical decision on real words and pseudowords are summed to obtain a total Decoding score (maximum accuracy score: 113).

#### 2.2.2 Fluency

Fluency, i.e., accurate and rapid orthography-to-phonology mappings, is assessed through a single text that the child is asked to read aloud. The text consists of 279 words and the examiner records the total number of words read correctly in 1 min. The child receives 1 point for each word s/he reads correctly (maximum accuracy score: 279).

#### 2.2.3 Morphosyntax

Morphosyntactic skills are assessed through four tests: production of verbs marked with appropriate person, number, tense and aspect marking; production of morphologically complex, or else compound words; sentence formulation with visual cue; and sentence formulation without visual cue.

In verb production, the child is asked to read aloud or silently 7 sentences and complete them with the verb in parenthesis after marking it with the appropriate person, tense and aspect feature (see example 1 below). The child receives 1 point for each correct answer (maximum accuracy score: 7).

(1) xtes ta peðia __ðio katapliktikes tenies (vlepo)
      yesterday the children __two awesome movies (watch).
      Target answer: iðan [watched_3P.PL.PAST.PERF]_) (P = person; PL = plural; PERF = perfective).

In the compound word production test, the child is asked to read aloud or silently 8 sentences and complete them with a compound word (noun, adjective or verb) derived from two items in a parenthesis. The child needs to also mark the noun and adjective with the appropriate number and case feature, while verbs need to be marked with the appropriate person, number, tense and aspect feature (see example 2 below). The child receives 1 point for each correct answer (maximum accuracy score: 8).

(2) ta peðia prosferane luluðia se ena ___γiγada. (skliros, karðia)
      the children offered flowers to a __giant (hard, heart).
      Target answer: slirokarðos [hardhearted].

The sentence formulation test with visual cue assesses the child’s syntactic skills, since the participant is asked to read aloud or silently rows of intermingled words and then arrange them in the correct order to form a syntactically correct sentence (e.g., drinking/Mary/newspaper/tea/the/while). The sentences were of graded complexity defined in terms of length, subordination and prepositional phrases. Each trial comes with a picture that visualizes the meaning of the target sentence. The test comprises 8 sentences, so the maximum accuracy score is 8. The sentence formulation test without visual cue is identical to the former test, however, the sentences were not accompanied by pictures and the trials were 4, so the maximum accuracy score was 4.

Testing in each of the four morphosyntactic tests discontinues when the child makes 3 consecutive errors. Scores in verb production, compound word, and the two sentence formulation tests are summed to obtain a total Morphosyntax score (maximum accuracy score: 27).

#### 2.2.4 Reading comprehension

The experimental procedure included two tests, namely, a semantic equivalence sentence test, and text comprehension.

In the semantic equivalence test, the child is asked to read silently or aloud four sets of five sentences, and identify the two sentences that share the same meaning in each set (see example 3). The sets were of graded difficulty in terms of sentence length and syntactic complexity. Testing discontinues when the child makes 3 consecutive errors. The maximum accuracy score is 4.

(3) (i) The city council met to decide about the building of the parks.
    (ii) The city council has decided about the building of a new park.
    (iii) A new park is not going to be built in our neighborhood.
    (iv) The building of a new park requires high funding.
    (v) A decision about the building of a new park has been made by the city council. (Target answer: ii, v).

The text comprehension test includes three short story texts (range of words: 97–127) belonging to the informational genre (“The hidden treasure,” “Alexander the Great,” “Maya civilization”). The child is asked to read each text silently or aloud. Each text is accompanied by seven multiple-choice questions, tapping into various basic-level and global comprehension skills, including recall of information that has been explicitly mentioned in the text, guessing the meaning of a low-frequent word, finding the main idea of the story, detecting information that does not match the global meaning of the text, inferring meaning within context, and attributing mental states to the story characters. The maximum accuracy score per text was 7 points, so maximum accuracy score was 21 points.

Scores in the semantic equivalence and text comprehension tests are summed to obtain a total Reading Comprehension score (maximum accuracy score: 25).

### 2.3 Data analysis plan

We first provide descriptive statistics for the performances of each group in the tests assessing each reading domain, i.e., decoding, fluency, morphosyntax, reading comprehension. We next ran one-way ANOVA analyses to assess between-group differences across tests. Next, to examine the effect of decoding, fluency and morphosyntax on each group’s performance in the reading comprehension test, we next ran linear mixed effects models. The predictors in the models were groups’ scores in the tests assessing decoding, fluency and morphosyntax, and the dependent measure was reading comprehension scores. Individual participants’ intercept was included in each model as correlated random effect. All statistical analyses were completed using R statistical software v.1.14.

## 3 Results

Table 2 below presents the groups’ mean performance scores in the tests comprising each reading subdomain, i.e., decoding, fluency, morphosyntax, and reading comprehension. We also present the percentile-equivalent scores based on the available norms of Test-A, and the number (and percentage) of children performing below the 10th percentile, between the 11th and the 30th percentile, and above the 31st percentile.

**Table 2:** Descriptive statistics (Means, *Standard Deviations*) of performance scores in the subdomains of Test-A, and percentile scores for the two groups of children with ASD.

Reading subdomain		Group A (*n* = 22)	Group B (*n* = 16)
Decoding	Pseudowords (max. accuracy score: 24)	15.1 (*5.7*)	13.7 (*6.8*)
	Real words (max. accuracy score: 53)	40.3 (*5.7*)	39.8 (*11.7*)
	Lexical decision (max. accuracy score: 36)	35.0 (*6.0*)	29.8 (*4.8*)
	max. accuracy score: 113	90.4 (*17.2*)	83.4 (*18.5*)
	Mean percentile	44^th^ (*22.3*)	48^th^ (*19.8*)
	N of children <10th percentile	3 (13.6%)	2 (12.5%)
	N of children between 11th and 30th percentile	3 (13.6%)	2 (12.5%)
	N of children >31st percentile	16 (72.8%)	12 (75.0)
Fluency	Fluency max. accuracy score: 279	72.6 (*19.4*)	58.7 (*19.4*)
	Mean percentile	24^th^ (*26.5*)	17^th^ (*20.2*)
	N of children <10th percentile	8 (36.3%)	8 (50.0%)
	N of children between 11th and 30th percentile	6 (27.4%)	4 (25.0%)
	N of children >31st percentile	8 (36.3%)	4 (25.0%)
Morphosyntax	Verb production (max. accuracy score: 7)	2.4 (*1.1*)	2.2 (*1.1*)
	Compound word production (max. accuracy score: 8)	3.6 (*2.3*)	1.5 (*1.2*)
	Sentence formulation with visual cue (max. accuracy score: 8)	5.7 (*2.3*)	5.8 (*1.9*)
	Sentence formulation without visual cue (max. accuracy score: 4)	2.4 (*1.3*)	1.1 (*0.9*)
	max. accuracy score: 27	14.0 (5*.4*)	10.5 (*2.6*)
	Mean percentile	43^th^ (*22.2*)	13^th^ (*10.6*)
	N of children <10th percentile	6 (27.3%)	5 (31.3%)
	N of children between 11th and 30th percentile	6 (27.3%)	8 (50.0%)
	N of children >31st percentile	10 (45.4%)	3 (18.7%)
Reading comprehension	Semantic equivalence (max. accuracy score: 4)	2.0 (*1.3*)	1.1 (*1.2*)
	Text comprehension (max. accuracy score: 21)	14.3 (*3.8*)	9.9 (*4.9*)
	max. accuracy score: 25	16.3 (*4.5*)	11.0 (*6.0*)
	Mean percentile	47^th^ (*28.6*)	23^th^ (*24.0*)
	N of children <10th percentile	1 (4.5%)	7 (43.8%)
	N of children between 11th and 30th percentile	6 (27.3%)	2 (12.4%)
	N of children >31st percentile	15 (68.2%)	7 (43.8%)

ASD = Autism Spectrum Disorder; Group A = children with ASD and intact cognitive abilities; Group B = children with ASD and low cognitive abilities; max. = maximum; *N* = number. The numbers in italics only in the parentheses indicate the Standard Deviations.

### 3.1 Decoding

The two groups did not differ significantly in the total Decoding accuracy scores, *F*(1, 36) = 1.296, *p* = 0.262, *η^2^* = 0.04. The two groups did not differ in either pseudoword, *F*(1, 36) = 0.157, *p* = 0.694, *η^2^* = 0.01, or real word reading, *F*(1, 36) = 0.039, *p* = 0.845, *η^2^* = 0.01. However, children with ASD and intact cognitive skills in Group A scored significantly higher than their peers with low cognitive skills in the lexical decision test, *F*(1, 36) = 8.081, *p* = 0.007, *η^2^* = 0.19.

### 3.2 Fluency

Children with ASD and intact cognitive abilities scored significantly higher than their peers with low cognitive skills in the Fluency test, *F*(1, 36) = 3.145, *p* = 0.049, *η^2^* = 0.10.

### 3.3 Morphosyntax

Children with ASD and intact cognitive abilities scored significantly higher than their peers with low cognitive skills in the total Morphosyntactic accuracy scores, *F*(1, 36) = 5.830, *p* = 0.021, *η^2^* = 0.14. The two groups did not differ in verb production, *F*(1, 36) = 0.227, *p* = 0.636, *η^2^* = 0.01. However, Group A scored higher than Group B in sentence formulation with visual cues, *F*(1, 36) = 10.465, *p* = 0.003, *η^2^* = 0.23, as well as in sentence formulation without visual cues, *F*(1, 36) = 10.063, *p* = 0.003, *η^2^* = 0.22. There was no Group effect in compound word production, *F*(1, 36) = 0.055, *p* = 0.975, *η^2^* = 0.00.

### 3.4 Reading comprehension

Children with ASD and intact cognitive abilities scored significantly higher than their peers with low cognitive skills in the total Reading comprehension accuracy scores, *F*(1, 36) = 9.094, *p* = 0.005, *η^2^* = 0.20. Group A scored significantly higher than Group B in both semantic equivalence, *F*(1, 36) = 4.629, *p* = 0.38, *η^2^* = 0.12, and text comprehension, *F*(1, 36) = 8.793, *p* = 0.005, *η^2^* = 0.19.

Tables 3, 4 below present the results of the linear mixed effects modes for the children with ASD and intact cognitive abilities (Group A) and their peers with low cognitive skills (Group B), respectively. For Group A, reading comprehension scores were significantly predicted by their scores in lexical decision, that was part of the Decoding subdomain, Fluency, and compound word production that was part of the Morphosyntax subdomain. For Group B comprising children with ASD and low cognitive skills, the model showed that their reading comprehension performance was independently driven by their pseudoword reading, lexical decision and verb production skills.

**Table 3:** Potential predictors of reading comprehension for children with ASD and intact cognitive skills (Group A).

	Estimate	SE	df	*t*-Value	*p* value
Intercept	10.56	5.936	0.938	1.780	0.33
Pseudoword reading	0.692	0.740	0.884	0.936	0.537
Real word reading	−6.319	6.327	14.399	−0.999	0.334
Lexical decision	4.505	1.299	5.242	3.886	0.007**
Total decoding scores	0.479	0.500	2.804	0.959	0.413
Fluency	0.088	0.067	1.338	4.303	0.004**
Verb production	6.516	6.369	0.886	1.023	0.509
Compound word production	1.529	1.141	0.908	2.340	0.04*
Sentence formulation with visual cue	1.863	1.351	0.800	1.378	0.438
Sentence formulation without visual cue	2.462	1.959	0.901	1.257	0.445
Total morphosyntactic scores	0.691	0.325	0.843	2.023	0.215

ASD = Autism Spectrum Disorder; SE = Standard Error; df = difference.

*p < 0.05.

**p < 0.01.

**Table 4:** Potential predictors of reading comprehension for children with ASD and low cognitive abilities (Group B).

	Estimate	SE	df	*t*-Value	*p* value
Intercept	5.123	2.759	19.000	1.857	0.079
Pseudoword reading	0.466	0.196	19.000	2.376	0.028*
Real word reading	0.349	0.180	2.429	1.941	0.169
Lexical decision	0.609	0.299	18.996	2.034	0.05*
Total decoding scores	0.206	0.091	0.004	2.254	0.983
Fluency	0.164	0.069	0.034	2.375	0.896
Verb production	2.516	1.123	19.000	2.241	0.037*
Compound word production	0.425	2.343	0.542	0.181	0.901
Sentence formulation with visual cue	0.392	0.530	19.000	0.740	0.468
Sentence formulation without visual cue	0.614	0.460	19.000	1.336	0.197
Total morphosyntactic scores	2.064	2.275	0.134	0.907	0.808

ASD = Autism Spectrum Disorder; SE = Standard Error; df = difference.

## 4 Discussion

The current study has examined the reading comprehension skills of two groups of age-matched school-aged children with ASD, one with intact and one with low cognitive abilities, and further investigated the way their reading comprehension performance is affected by their decoding, fluency and morphosyntactic skills. We found that the group with ASD and low cognitive skills exhibited significantly lower reading comprehension scores than their peers with intact cognitive abilities, and that the former group had moderate reading comprehension difficulties on the basis of the norms and cut-off values of the standardized reading assessment tool of the study. Furthermore, the reading comprehension performance of the group with ASD and intact cognitive abilities was found to be independently driven by their fluency skills, their ability to decide about the real word status of lexical items, and to a lesser extent, by their compound word productions skills. On the other hand, the children with ASD and low cognitive skills were found to rely on their pseudoword reading and verb production skills, and to a lesser extent, on their lexical decision ability, while performing in reading comprehension. The findings of the study show, first, that low intellectual skills may have a negative effect on children’s reading comprehension performance. Second, the presence of language impairment in children with ASD seems to also influence the mechanisms available to them to cope with reading comprehension difficulties. Finally, the study highlights that some children on the spectrum with intact cognitive abilities (about 32% according to the study’s findings) still face moderate-to-severe reading comprehension difficulties, further implying that ASD may influence reading comprehension ability regardless of normal intellectual functioning.

Specifically, the children with ASD and low cognitive skills performed above cut off in the Decoding subdomain only, while the group with intact cognitive skills performed above cut off in all reading subdomains except for fluency in which they exhibited moderate difficulties. The group with ASD and low cognitive skills scored lower than their peers with intact cognitive skills in almost all reading tests, except for verb production and compound word formation, word and pseudoword decoding. The finding that word decoding was a strength for the majority of the children with low cognitive skills seems to agree with previous research showing a selective preservation of phonological/orthographic code mappings co-occurring with poor comprehension when reading, which characterizes 20–35% of individuals with ASD (Klin et al., 2007; Meilleur et al., 2015). The strong dissociation observed between the children’s largely preserved decoding skills (75% of the children with ASD and low cognitive skills scored above the critical cut off for impairment in Decoding) and their moderately-to-severely impaired reading comprehension abilities (more than 55% of the children scored below the critical cut off for impairment in Reading comprehension) implies an asymmetry between their word recognition skills and their ability to assign meaning to what they read. Importantly, about 31% of the children with ASD and intact cognitive skills also scored below cut off for reading comprehension which suggests that reading comprehension is often vulnerable for children on the spectrum regardless of their intellectual functioning levels.

Interestingly, besides text comprehension, the group with ASD and low cognitive skills fell behind their peers with intact cognitive skills in the semantic equivalence test as well, which implies that comparing meaning across single sentence contexts was negatively affected by low intellectual functioning skills. So far, reading comprehension challenges in ASD have been assessed through texts whose understanding heavily relies on basic-level language mechanics, including semantics and morphosyntax, as well as higher order skills, including logical reasoning, inferencing and situation model building (Williamson et al., 2012). The semantic equivalence test in the current study mainly tapped into children’s ability to compare short sentences and decide on a pair of sentences conveying the same meaning, which involves metalinguistic decision-making. Research suggests that children with ASD exhibit difficulties in metalinguistic ability, or the ability to understand language outside the concrete meaning of words, relative to their TD peers (Lewis et al., 2007; Lucas and Norbury, 2014; Peristeri et al., 2021). The finding that the children with ASD and low cognitive skills in the current study scored lower than their peers with intact cognitive skills in the semantic equivalence test suggests that metalinguistic difficulties may be specific to those children with impaired cognitive functioning and may burden children tasked with retrieving meaning from sentential context.

Another aim of the study was to identify the factors influencing reading comprehension performance in each group. The findings showed a dissociation between the two groups in that the children with ASD and intact cognitive skills drew on their fluency and decoding skills, as well as their morphosyntactic resources to cope with reading comprehension, however, their peers with low cognitive skills tended to rely mostly on their decoding skills (specifically, pseudoword reading), and to a lesser extent on their lexical decision and morphosyntactic skills. Specifically, the strongest predictor of the former group’s reading comprehension performance was fluency defined in the literature as accurate, rapid, expressive oral reading that reflects a reader’s efficiency to connect the printed word with its syntactic and meaning aspects (Jenkins and O’Connor, 2003; Pikulski and Chard, 2005; Klauda and Guthrie, 2008). Out of the decoding measures that have been used in the current study, lexical decision, i.e., identifying real words from pseudowords, was the only measurement that was found to drive the reading comprehension performance of the children with ASD and intact cognitive skills. Specifically, those children with intact cognitive skills that exhibited higher tendency to accept pseudowords as real in the lexical decision test performed worse in reading comprehension as compared to their peers with intact cognitive skills who exhibited better ability to identify pseudowords. We should note that the specific test mainly assesses the child’s ability to make an explicit decision on whether or not a letter string is a word, which engages the individual in some level of metalinguistic analysis, by-passing more automatized response routes, such as those involved in rapid recognition of single words (Gold and Rastle, 2007). The fact that the ability of the children with ASD and intact cognitive skills to read words and pseudowords had no significant contribution to their reading comprehension performance, in contrast to lexical decision which had a significant role in comprehension, suggests that metalinguistic, controlled processes were more relevant to meaning integration processes in reading comprehension than automatic processes of mapping orthographic to phonological representations as reflected in single word reading/decoding. This aligns with prior research positing that lexical decision tasks necessitate the engagement of metalinguistic and controlled cognitive processes (Rastle and Brysbaert, 2006; Verhoeven and Perfetti, 2011). The finding that metalinguistic judgment was relevant to the reading comprehension skills of the children with ASD and intact cognitive skills over and above decoding is important since it suggests that their ability to extract meaning from written input depended on their metacognitive ability, such as semantic memory skills, which have been found to be preserved in high-functioning individuals (Wojcik et al., 2013). Future research directions can investigate this further by exploring the role of metalinguistic skills of children with ASD in their reading comprehension performance.

Compound word formation was another factor that was found to contribute to reading comprehension in the group with ASD and intact cognitive skills. Children with higher production of compound words performed better in reading comprehension as compared to their peers with lower compound word production rates. As Greek is a highly inflected language, compound words need to be marked with grammatical features, like case, gender, and number (in case the building blocks of a compound are nouns or adjectives), and person, number and tense in case of verb compounds. Errors in the compound word test of the current study resulted from either erroneous grammatical marking of the compound, e.g., skirokarrðia_FEMININE_ γiγada_MASCULINE_ ‘hardhearted giant’, wherein the child failed to mark the compound adjective with the correct grammatical gender feature in order for the adjective to agree with the following masculine noun, or, even more markedly than erroneous grammatical marking, the inappropriate placement of stress, e.g., γinekopeðά ‘women and children’, instead of γinekόpeða. According to Tsiamas et al. (2015), compound formation in Greek is a demanding process in that it taps into both morphosyntactic and phonological knowledge to decide upon the gender class and the stress position of the compound word, which do not always coincide with the gender or/and the stress position of the compound’s building blocks. The texts included in the reading comprehension subtest of the current study included a high number of morphologically compound words (e.g., kiniγoskilo ‘hunting dog’, filosofos ‘philosopher’), especially since compounding in Greek is very common (Tsesmeli and Koutselaki, 2012). The frequent use of compound words in the texts has probably contributed to the fact that the particular test had a significant contribution to the children’s reading comprehension performance. We should note that the performance of the group with ASD and low cognitive abilities in compound word production was lower than that of the children with intact cognitive skills, which agrees with many studies that have found morphosyntactic difficulties in children on the spectrum (Eigsti et al., 2007; Sukenik and Friedmann, 2018; Peristeri et al., 2023).

The strength of the children with ASD and low cognitive skills in decoding appeared to have the most significant impact on their reading comprehension performance. In fact, decoding effects on reading were mainly driven from the children’s pseudoword reading efficiency which did not differ from their peers with intact cognitive skills, further implying that the phonological reading strategies of the children with ASD and low cognitive skills were preserved and, thus, boosted their reading comprehension performance. Besides decoding, verb production significantly predicted Group B’s reading comprehension performance, while lexical decision (a proxy for metalinguistic skills) also influenced their reading performance, though to a lesser extent as compared to their peers with intact cognitive functioning skills.

The results of the current study should be interpreted in the context of several limitations. First, the clusters’ sample sizes, especially for the group with ASD and low cognitive skills, limit the interpretations of our findings on reading comprehension and the way it was affected in each group, so larger samples are needed to verify our results. Also, there are many studies showing that reading comprehension is affected by children’s executive functions and theory of mind (Guajardo and Cartwright, 2016; Butterfuss and Kendeou, 2018; Andreou et al., 2022; Dicataldo et al., 2023), which have not been considered in the present study and should be investigated to gain full knowledge of the factors underlying reading difficulties in children on the spectrum.

In conclusion, the overall findings of the study suggest that reading comprehension is challenging for children on the spectrum, especially the ones with low cognitive abilities. While children with ASD and intact cognitive skills managed to cope with reading comprehension by drawing on their lexical decision, fluency and morphosyntactic skills, their peers with low cognitive abilities mainly relied on their decoding competence to compensate for their reading comprehension difficulties. Importantly, most of the resources employed by the children with ASD, especially the ones with intact cognitive skills (e.g., identifying real words from pseudowords, identifying semantically equivalent sentences) engaged children in metalinguistic reasoning, which seemed to be important in moderating their reading comprehension difficulties. Further research is needed to investigate the relationship between metalinguistic ability and the reading performance of children with ASD. The findings of the study are novel in that they highlight reading comprehension difficulties and the reasons behind them in children with ASD and low cognitive abilities that are underrepresented in autism research. Further behavioral studies in reading in these children are warranted to further investigate the origins of their academic underachievement and to inform intervention designs and targets for these individuals. Also, neuroimaging studies investigating the neural correlates of reading comprehension in ASD could shed more light in the causes of reading comprehension difficulties of children on the spectrum with intact or low cognitive skills.

## Data availability statement

The raw data supporting the conclusions of this article will be made available by the authors, without undue reservation.

## Ethics statement

The studies involving humans were approved by Aristotle University of Thessaloniki Institutional review board (IRB) (IRB protocol number: 39928). The studies were conducted in accordance with the local legislation and institutional requirements. Written informed consent for participation in this study was provided by the participants’ legal guardians/next of kin.

## Author contributions

EP: Conceptualization, Methodology, Project administration, Writing – original draft, Writing – review & editing. CF: Formal analysis, Writing – review & editing. MA: Conceptualization, Methodology, Writing – original draft, Writing – review & editing.
